# Apoptosis as an evolutionary battleground: pathogen pressure and the shaping of programmed cell death pathways

**DOI:** 10.3389/fcell.2026.1800300

**Published:** 2026-06-12

**Authors:** Fiordaliso Carolina Román-Carraro, Laila Gutiérrez-Kobeh

**Affiliations:** 1 Unidad de Investigación UNAM-INC, División de Investigación, Facultad de Medicina, Universidad Nacional Autónoma de México-Instituto Nacional de Cardiología “Ignacio Chávez”, Mexico City, Mexico; 2 Departamento de Procesos y Tecnología, Universidad Autónoma Metropolitana-Cuajimalpa, Ciudad de, México, México

**Keywords:** apoptosis, Bcl-2 family, caspases, coevolution, host–pathogen interactions, innate immunity, mitochondria, regulated cell death

## Abstract

Apoptosis is a tightly regulated form of programmed cell death that enables the controlled elimination of damaged or infected cells without eliciting deleterious inflammatory responses. Beyond its fundamental roles in embryogenesis, tissue homeostasis, and cellular turnover, the molecular architecture of apoptosis reflects deep evolutionary origins shaped by mitochondrial quality control, the emergence of intercellular communication, and immune surveillance mechanisms. Apoptotic signaling is initiated through three principal pathways, the extrinsic (death receptor–mediated), perforin/granzyme-mediated, and intrinsic (mitochondrial) pathways, which converge on caspase activation as the final execution step. Accumulating evidence indicates that persistent interactions with intracellular pathogens have profoundly influenced the evolution and diversification of these pathways. Viruses, bacteria, fungi, and protozoan parasites have independently evolved convergent strategies to suppress, delay, redirect, or exploit apoptosis by targeting conserved regulatory nodes, including mitochondrial outer membrane permeabilization, Bcl-2 family proteins, Bid-mediated pathway integration, Apaf-1–dependent caspase activation, and inhibitors of apoptosis proteins. These pathogen-driven pressures have not only shaped infection outcomes but have also contributed to the expansion, redundancy, and regulatory complexity of host apoptotic machinery. Here, we synthesize recent advances in the understanding of pathogen-mediated modulation of apoptosis and propose that programmed cell death operates as part of an integrated, evolutionarily conserved network of host defense. In this framework, apoptosis emerges as a central battleground in host–pathogen coevolution, linking cellular homeostasis to immune protection.

## 1 Introduction

Microbes and humans establish interactions that can be either beneficial or harmful to human health. Organisms that cause disease are known as pathogens (Casadevall and Pirofski, 1999) and include a wide range of taxa, from unicellular such as bacteria, protozoa, and fungi to multicellular parasites such as helminths. Viruses represent a special case: although not considered living organisms, they include highly pathogenic agents, as illustrated by the emergence of SARS-CoV-2. In addition, many infectious diseases with pandemic potential originate from zoonotic pathogens transmitted to humans through close and sustained contact with animal reservoirs (Balloux and van Dorp, 2017). Throughout evolution, hosts and pathogens have interacted dynamically, with hosts developing mechanisms to detect and eliminate infection, while pathogens evolve strategies to evade immune responses and manipulate host cellular processes to promote replication and spread (Jo, 2019). This reciprocal selective pressure has shaped not only immune defenses but also fundamental cellular regulatory pathways, including apoptosis (Kaczanowski, 2016).

Apoptosis is a widely conserved defense mechanism in multicellular organisms and represents an active form of cell death induced by developmental signals and cellular stress. During apoptosis, dying cells are dismantled in an orderly manner and removed with minimal inflammatory signaling. Importantly, apoptosis also functions as a cell-intrinsic defense mechanism that eliminates infected or damaged cells, thereby limiting pathogen propagation (Häcker, 2018). From an evolutionary standpoint, the ability of infected cells to undergo apoptosis is considered an early form of innate immunity that predates the emergence of adaptive immune responses (Bolnick et al., 2025). In response to this selective pressure, many pathogens have evolved mechanisms to interfere with apoptotic signaling, allowing them to complete their life cycles within host cells (Best, 2008; Nichols et al., 2017; Ito and Orth, 2012).

Apoptosis is a form of regulated cell death (RCD) characterized by a non-lytic process operating under both physiological and pathological conditions. It involves coordinated intrinsic and extrinsic signaling cascades that converge on caspase activation and controlled cellular dismantling. While apoptosis was traditionally defined by morphological features, current guidelines emphasize biochemical and molecular criteria as the primary basis for classification (Galluzzi et al., 2015; Galluzzi et al., 2018; Tang et al., 2019). Recent comprehensive reviews further highlight the integration of intrinsic and extrinsic pathways and their relevance to disease and host defense (Mustafa et al., 2024). In vertebrates, cytotoxic immune cells also induce apoptosis through the perforin–granzyme pathway, representing a specialized immune-mediated mechanism for eliminating infected or transformed cells (Chowdhury and Lieberman, 2008; Voskoboinik et al., 2015).

Stress-activated signaling pathways integrate environmental and immune cues into apoptotic decisions. Among these, the p38 mitogen-activated protein kinase (p38 MAPK) pathway functions as a central stress-response module activated by inflammatory cytokines, oxidative stress, DNA damage, and microbial infection (Arthur and Ley, 2013; Cuadrado and Nebreda, 2010). p38 signaling promotes apoptosis through transcriptional and non-transcriptional mechanisms, including regulation of p53 activity, modulation of Bcl-2 family proteins, and induction of mitochondrial outer membrane permeabilization (Wagner and Nebreda, 2009; Aubrey et al., 2018). Recent work demonstrates that pathogen-derived signals activate MAPK and NF-κB pathways during infection, directly linking innate immune signaling to apoptotic outcomes (Shen et al., 2024). From an evolutionary perspective, the incorporation of p38 MAPK into apoptotic regulation illustrates how ancient stress-sensing pathways were refined under pathogen-driven selective pressure (Kalapos et al., 2019; Cánovas and Nebreda, 2021).

Accumulating evidence indicates that the molecular machinery governing apoptosis is ancient and conserved across metazoans (Lasi et al., 2010; Horkan et al., 2024). Core apoptotic components, including caspase-like proteases, Bcl-2 family regulators, and apoptosome-associated adaptors, derive from ancestral stress-response and mitochondrial quality-control systems that were progressively refined during the evolution of multicellular life (Kaczanowski, 2016; Kaushal et al., 2023).

Over the past decade, multiple studies have emphasized that apoptosis rarely acts in isolation during infection, but instead intersects with other regulated cell-death pathways, forming an integrated host-defense network (Ruan et al., 2024; Eskander et al., 2025). In parallel, pathogens have evolved the capacity to suppress, delay, redirect, or overstimulate apoptosis depending on their replication strategies, generating an evolutionary arms race between host defenses and pathogen survival mechanisms (Rodríguez-González and Gutiérrez-Kobeh, 2023).

At the molecular level, emerging work has revealed additional regulatory layers in death receptor signaling and their relevance for apoptotic checkpoint control (Seyrek et al., 2020), while a growing body of evidence underscores the integration of apoptosis with other regulated cell death programs during infection, including PANoptosis and coordinated inflammatory cell death pathways (Guo et al., 2025; He et al., 2025).

## 2 Apoptosis and the p38 pathway

### 2.1 Discovery of apoptosis as a regulated biological process

Apoptosis was established as a regulated, genetically encoded form of cell death by Kerr, Wyllie, and Currie, who described it as a process essential for tissue homeostasis, development, and disease (Kerr et al., 1972). Their work demonstrated that cell death is not merely a passive consequence of injury but an intrinsic biological program, laying the foundation for genetic and molecular studies that revealed the evolutionary conservation of apoptotic mechanisms across metazoans.

Genetic analyses in *Caenorhabditis elegans* defined the core components of the intrinsic apoptotic machinery. Under basal conditions, the Bcl-2 homolog CED-9 binds and inhibits the Apaf-1 ortholog CED-4, thereby preventing activation of the initiator caspase CED-3. Upon apoptotic signaling, the BH3-only protein EGL-1 is induced and disrupts the CED-9–CED-4 complex, releasing CED-4, which oligomerizes into an apoptosome-like structure that promotes CED-3 activation and execution of apoptosis (Horvitz, 1999; Lettre and Hengartner, 2006; Conradt and Horvitz, 1998; Del Peso et al., 1998). The functional conservation of this pathway became evident with the identification of mammalian homologs, including Bcl-2, Apaf-1, and caspase-9, which together regulate mitochondrial outer membrane permeabilization (MOMP) and apoptosome assembly in vertebrate cells. Comparative and functional studies demonstrated that expression of *C. elegans* apoptotic components can trigger cell death when ectopically expressed in mammalian systems, highlighting the deep evolutionary conservation of the apoptotic program (Chinnaiyan et al., 1997; Bratton and Salvesen, 2010).

### 2.2 p38 MAPK as an ancient integrator of stress, immunity, and apoptosis

Comparative analyses indicate that the p38 MAPK pathway is an ancient stress-response module that emerged early in opisthokont evolution and later diversified in metazoans, becoming closely associated with innate immune signaling (Shabardina et al., 2023). In the context, p38 is relevant because it links infection-associated stress signals with apoptotic outcomes. During pathogen infection, p38 is activated by pathogen-derived molecules, inflammatory cytokines, oxidative stress, and DNA damage, integrating these inputs to regulate antimicrobial gene expression, cellular metabolism, and apoptosis. Rather than acting as a peripheral stress kinase, p38 contributes to the decision of whether infected cells activate apoptosis or maintain viability. This positioning makes p38 a frequent target of pathogen interference, highlighting its importance in host–pathogen coevolution. By embedding p38 within apoptotic regulation, apoptosis can be viewed as a coordinated response to infection and stress rather than an isolated cell-autonomous process (Arthur and Ley, 2013; Kaczanowski, 2016; Shabardina et al., 2023).

### 2.3 Conservation and diversification of apoptotic pathways

The apoptotic framework first characterized in *C. elegans* is conserved in mammals, where BH3-only proteins inhibit anti-apoptotic Bcl-2 family members, enabling Apaf-1–dependent apoptosome assembly and caspase-9 activation. In metazoans, this ancestral system diversified into intrinsic (mitochondrial) and extrinsic (death receptor–mediated) pathways. The intrinsic pathway, dependent on Bcl-2 family proteins, Apaf-1, and caspase-9, is broadly conserved and considered the more ancient of the two, closely linked to mitochondrial biology and endosymbiosis (Fuchs and Steller, 2011; Shabardina et al., 2023).

**FIGURE 1:**
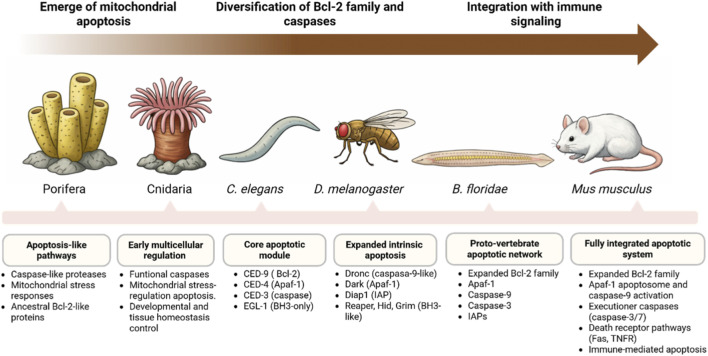
Evolutionary diversification of apoptotic pathways across metazoans. Apoptosis has deep evolutionary roots, tracing back to ancient mitochondrial stress–response mechanisms already present in early-branching animals. In Porifera and Cnidaria, apoptosis-like pathways operate through caspase-like proteases and mitochondrial stress–regulated cell death, supporting basic tissue remodeling and homeostasis. In *Caenorhabditis elegans*, apoptosis is regulated by a minimal, highly conserved core module composed of CED-9, CED-4, CED-3, and EGL-1. In arthropods such as *Drosophila melanogaster*, apoptotic regulation expanded through inhibitor of apoptosis protein (IAP)–controlled caspase networks. In the cephalochordate *Branchiostoma floridae*, an expanded yet non-redundant repertoire of Bcl-2 family members, caspases, and Apaf-1 reflects a proto-vertebrate apoptotic architecture. In vertebrates, represented here by *Mus musculus*, apoptosis diversified into intrinsic, extrinsic, and immune-mediated pathways, integrating mitochondrial control with immune surveillance. Together, this evolutionary trajectory illustrates how apoptosis gradually diversified under selective pressure, giving rise to the complex, immune-integrated death networks observed in modern vertebrates.

In vertebrates, apoptosis acquired an additional immune-mediated mechanism through the granzyme–perforin system used by cytotoxic T lymphocytes and natural killer cells. Perforin enables the delivery of granzymes into target cells, where they trigger caspase-dependent and caspase-independent apoptotic pathways. Evolutionary analyses indicate that this system is restricted to jawed vertebrates and emerged alongside adaptive immunity, representing a late innovation that complements ancestral stress- and mitochondria-dependent apoptotic mechanisms (Figure 1) (Chowdhury and Lieberman, 2008; Voskoboinik et al., 2015; Flajnik and Kasahara, 2010).

Finally, apoptosis-like processes have also been reported in unicellular eukaryotes, including yeast and protozoa, where several proteins share sequence or functional similarities with metazoan apoptotic regulators. Although their biological significance remains debated, these observations support the view that ancient stress-response pathways were later adapted and refined to regulate apoptosis in multicellular organisms (Figure 1) (Lockshin and Zakeri, 1994; Gordeeva et al., 2004; Kulkarni and Hardwick, 2023; Tummers and Green, 2022).

## 3 Initiators and executors of apoptosis activation

The progression of apoptosis involves two tightly coordinated phases: initiation and execution. The initiation phase entails the activation of specific genes and signaling pathways that commit the cell to death, ultimately leading to the execution phase, during which caspases (aspartate-specific, cysteine-dependent peptidases) play a central role. Initiator caspases, most prominently caspase-8 and caspase-9, and in more context-dependent settings caspase-2 and caspase-10, sense apoptotic signals and activate executioner caspases, including caspase-3, caspase-6, and caspase-7 (Taylor et al., 2008; Riedl and Shi, 2004; Kumar, 2007). These executioner caspases mediate the hallmark features of apoptosis, such as DNA fragmentation, proteolytic cleavage of nuclear and cytoskeletal proteins, membrane blebbing, apoptotic body formation, and the regulated exposure of phagocytic “eat-me” signals, ensuring efficient clearance of dying cells without inflammation (Taylor et al., 2008; Nagata et al., 2010; Dho et al., 2025).

Apoptotic signaling can be initiated through three major, partially overlapping pathways: the extrinsic (death receptor–mediated) pathway, the intrinsic (mitochondrial) pathway, and the perforin/granzyme pathway employed by cytotoxic lymphocytes. Rather than operating as independent routes, these pathways are highly interconnected and converge at the level of executioner caspase activation through multiple amplification and feedback loops (Taylor et al., 2008; Eskander et al., 2025). This convergence underscores apoptosis as an integrated cellular program in which stress sensing, immune effector mechanisms, and mitochondrial signaling are coordinated to ensure irreversible commitment to cell death, consistent with its evolutionary role as a core component of host defense.

## 4 Three pathways initiate apoptosis in mammals (revised and updated)

### 4.1 The intrinsic pathway

The intrinsic apoptotic pathway is activated by intracellular stress signals such as DNA damage, oxidative stress, endoplasmic reticulum stress, hypoxia, radiation, and nutrient deprivation. Although diverse in origin, these signals converge at the mitochondria, where mitochondrial outer membrane permeabilization (MOMP) represents the irreversible commitment step of apoptosis. MOMP is mediated by the oligomerization of the pro-apoptotic Bcl-2 family members Bax and Bak, which form pores in the outer mitochondrial membrane and enable the release of apoptogenic factors (Youle and Strasser, 2008; Green and Llambi, 2015).

Following MOMP, cytochrome c is released into the cytosol and binds APAF-1, promoting apoptosome assembly and activating caspase-9, which, in turn, activates executioner caspases such as caspase-3 and caspase-7. Additional mitochondrial proteins, including SMAC/DIABLO, HtrA2/Omi, apoptosis-inducing factor (AIF), and endonuclease G (EndoG), amplify apoptotic signaling or mediate caspase-independent cell death (Candé et al., 2004; Vucic et al., 2011).

MOMP functions as a central integration point for stress, metabolic, and immune-derived signals. Recent studies indicate that mitochondrial dynamics, redox balance, and mitophagy strongly influence apoptotic sensitivity during infection, linking intrinsic apoptosis to pathogen-induced metabolic and inflammatory stress (Chen et al., 2023; Abbas and Ghssein, 2025). These findings support the view that mitochondrial apoptosis has been repeatedly shaped by infectious pressure, acting as an early cell-intrinsic defense mechanism against intracellular pathogens.

From an evolutionary perspective, the central role of mitochondria in apoptosis reflects their endosymbiotic origin. Core components of the intrinsic pathway likely derive from ancestral bacterial toxin-like systems that were domesticated during early eukaryotic evolution and subsequently refined under selective pressures associated with multicellularity and infection (Kaushal et al., 2023). Although intrinsic apoptosis is deeply conserved across metazoans, lineage-specific modifications in regulatory architecture suggest ongoing adaptation to distinct pathogen landscapes (Galluzzi et al., 2018; Krasovec et al., 2023).

### 4.2 The extrinsic pathway

The extrinsic apoptotic pathway is initiated by extracellular ligands binding to cell-surface death receptors, primarily members of the tumor necrosis factor receptor (TNFR) superfamily (Zhang et al., 2022). These receptors contain intracellular death domains that recruit adaptor proteins such as FADD or TRADD, leading to assembly of the death-inducing signaling complex (DISC) and activation of initiator caspase-8 (Ashkenazi and Dixit, 1998; Huyghe et al., 2023).

TNFR1 signaling illustrates how death receptor pathways integrate survival and apoptotic decisions. Early formation of membrane-associated Complex I promotes NF-κB-dependent transcription of pro-survival genes. In contrast, delayed formation of cytosolic Complex II enables caspase-8 activation and apoptosis when survival signaling fails (Green and Llambi, 2015). Regulation of this balance by cellular inhibitors of apoptosis (c-IAPs) and ubiquitination of RIPK1 allows dynamic switching between survival, apoptosis, and necroptosis (Varfolomeev et al., 2007; Amusan et al., 2025).

From an evolutionary standpoint, the extrinsic pathway appears to be more recent than mitochondrial apoptosis and is primarily restricted to vertebrates. Canonical death receptor–mediated apoptosis is absent in classical invertebrate models, although partial conservation of caspase-8- and FADD-like proteins has been identified in basal deuterostomes (Zmasek and Godzik, 2013; Horkan et al., 2024). Recent comparative genomics studies suggest that death receptor signaling evolved through the gradual assembly of pre-existing death-domain modules, likely driven by increasing immune complexity and pathogen-mediated selective pressure in early vertebrates (Kandouz, 2024; Zhang et al., 2025). Beyond canonical ligand–receptor interactions, additional regulatory layers operate at the level of death receptors themselves. Recent evidence indicates that glycosylation patterns modulate receptor clustering, signaling strength, and downstream apoptotic commitment, positioning glycans as functional checkpoints in extrinsic pathway activation (Seyrek et al., 2025).

### 4.3 The perforin/granzyme pathway

Cytotoxic lymphocytes, including natural killer (NK) cells and CD8^+^ T lymphocytes, eliminate infected or transformed cells through the perforin/granzyme pathway. Upon target recognition, perforin forms pores in the target cell membrane, allowing granzymes to enter the cytosol and initiate cell death (Martinvalet, 2019).

Granzyme B induces apoptosis by directly activating executioner caspases and cleaving Bid, thereby engaging mitochondrial amplification of cell death. Granzyme A primarily triggers caspase-independent pathways associated with mitochondrial dysfunction and DNA damage. In humans, granulysin further contributes by inducing microptosis, a programmed death pathway specialized for eliminating intracellular pathogens (Dotiwala et al., 2016).

Recent studies demonstrate that granzymes can also activate inflammatory forms of regulated cell death by cleaving gasdermins, linking cytotoxic lymphocyte activity to pyroptosis and immunogenic cell death. This mechanism is particularly relevant during viral infection and tumor immunity, where pathogen- or tumor-driven immune pressure shapes the mode of target cell elimination (Zhou et al., 2020; de Miguel et al., 2022; Pasparakis and Vandenabeele, 2015).

Collectively, the perforin/granzyme pathway illustrates how immune-mediated apoptosis and inflammatory cell death have co-evolved as adaptive responses to persistent pathogen pressure, complementing mitochondrial and death receptor–mediated apoptotic programs. Post-transcriptional regulation adds an additional layer of control over apoptotic commitment, with specific microRNAs fine-tuning caspase activation and mitochondrial signaling pathways (Chen et al., 2025).

**FIGURE 2:**
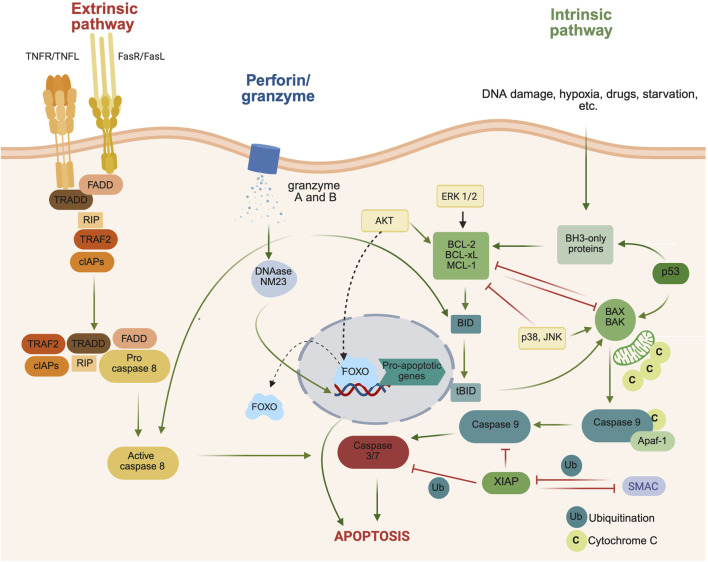
Integration of extrinsic, intrinsic, and immune-mediated apoptotic pathways. Apoptosis is initiated through three major signaling routes that converge on the activation of executioner caspases. The extrinsic pathway is triggered by death receptor engagement, including TNFR/TNFL and FasR/FasL, leading to recruitment of adaptor proteins (TRADD, FADD, RIP, TRAF2) and formation of the death-inducing signaling complex (DISC), which activates caspase-8. Caspase-8 directly activates executioner caspases and links the extrinsic pathway to the mitochondrial pathway through BID cleavage. The intrinsic pathway is activated by intracellular stress signals such as DNA damage, hypoxia, drug exposure, and nutrient deprivation. These stimuli induce BH3-only proteins and p53, promoting activation of BAX and BAK, mitochondrial outer membrane permeabilization, cytochrome c release, apoptosome assembly (Apaf-1 and caspase-9), and subsequent activation of caspases-3 and -7. In parallel, immune cytotoxicity mediated by perforin and granzymes provides an additional route for direct activation of apoptotic signaling. Cell fate is finely regulated by pro-survival pathways, including AKT, ERK1/2, and NF-κB, as well as by inhibitor of apoptosis proteins (IAPs) and their antagonist SMAC. Together, these interconnected networks integrate extracellular cues, intracellular stress, and immune surveillance to determine the balance between cell survival and programmed cell death.

Together, the intrinsic, extrinsic, and perforin/granzyme pathways demonstrate that apoptosis is a regulated, interconnected system whose key nodes have been shaped by long-term host–pathogen interactions (Figure 2).

## 5 Central participants in apoptosis

### 5.1 Mitochondria

Mitochondria are essential eukaryotic organelles that perform two fundamental functions: ATP synthesis via oxidative phosphorylation and regulation of programmed cell death (PCD). In intrinsic apoptosis, mitochondria act as central decision-makers by integrating intracellular stress signals and executing mitochondrial outer membrane permeabilization (MOMP), a commitment step mediated by the oligomerization of the pro-apoptotic Bcl-2 family proteins Bax and Bak. MOMP facilitates the release of apoptogenic factors, including cytochrome c, SMAC/DIABLO, HtrA2/Omi, AIF, and EndoG, which activate either caspase-dependent pathways via apoptosome formation or caspase-independent pathways through nuclear DNA fragmentation, ensuring controlled cell dismantling with minimal inflammation (Green and Llambi, 2015; Tait and Green, 2013).

The apoptotic machinery is thought to have originated from the ancient endosymbiosis between a proto-eukaryotic host and an α-proteobacterium that eventually evolved into mitochondria. Phylogenomic and structural analyses indicate that core components of apoptosis, including Bcl-2 family proteins and several mitochondrial proteases, share homology with bacterial toxin–antitoxin systems, suggesting that mitochondrial ancestors may have used these proteins in conflict-defense interactions that were later co-opted into regulated cell death pathways in eukaryotes (Kaczanowski, 2016; Kaushal et al., 2023). This process, often described as mitochondrial domestication, reflects the evolutionary transition from antagonistic host–symbiont interactions to cooperative cellular integration.

The emergence of multicellularity further reinforced the requirement for regulated elimination of damaged or superfluous cells, making apoptosis essential for tissue integrity and developmental patterning across metazoan lineages (Kaushal et al., 2023). Notably, mitochondrial respiration and apoptosis remain tightly interconnected in extant organisms: respiratory activity modulates apoptotic sensitivity through reactive oxygen species (ROS) levels, metabolic flux, and regulation of the Bcl-2 family, while disruptions in apoptotic pathways can reciprocally alter oxidative phosphorylation (Green and Llambi, 2015; Tait and Green, 2013). Consistent with this view, experimental evidence from unicellular and multicellular models, including yeast, indicates that perturbations in apoptotic regulation lead to measurable alterations in mitochondrial respiration, underscoring the deep co-evolutionary coupling between bioenergetics and regulated cell death mechanisms.

### 5.2 Caspases: key molecules regulating apoptosis and inflammation

Caspases are a family of cysteine-dependent proteases that constitute the core execution and regulatory machinery of apoptosis and several inflammatory forms of regulated cell death. All caspases share a conserved catalytic fold, known as the caspase or hemoglobinase fold, which is also present in evolutionarily related protease families such as metacaspases, paracaspases, legumains, and gingipains (Aravind and Koonin, 2002; Poreba et al., 2013). Caspases are synthesized as inactive zymogens (procaspases) and require proteolytic processing for activation, ensuring tight temporal and spatial control of cell death decisions (Riedl and Shi, 2004; Dho et al., 2025).

#### 5.2.1 Functional classification and regulatory roles

In mammals, caspases are commonly classified into three functional groups based on their primary roles, although functional overlap and context dependency are increasingly recognized (Li et al., 2025).

Inflammatory caspases, including caspases-1, -4, and -5 (and murine caspase-11), function as cytosolic sensors of microbial components and danger signals. Their activation promotes cleavage of gasdermin D and execution of pyroptosis, linking pathogen detection to inflammatory cell death and innate immune amplification (Rathinam et al., 2019; Dai et al., 2023; Eckhart and Fischer, 2024).

Executioner caspases (caspases-3, -6, and -7) are responsible for the proteolytic dismantling of cellular structures during apoptosis, mediating DNA fragmentation, cytoskeletal breakdown, and membrane remodeling (Taylor et al., 2008; Dho et al., 2025).

Initiator caspases (caspases-2, -8, -9, and -10) respond to upstream death signals and activate executioner caspases through adaptor-mediated proximity-induced activation within multiprotein platforms such as the apoptosome or the death-inducing signaling complex (DISC) (Riedl and Shi, 2004; Prasanth Kumar et al., 2025).

This classification is not absolute. Caspase-2, for example, exhibits biochemical and functional properties that overlap with executioner caspases and plays a role in cellular stress surveillance rather than classical death receptor signaling (Julien and Wells, 2017; Svandova et al., 2024). Similarly, caspases traditionally associated with inflammation can influence apoptotic outcomes, underscoring extensive crosstalk between apoptotic and inflammatory pathways (Nadendla et al., 2025).

#### 5.2.2 Regulation of caspase activity

Given their potent proteolytic capacity, caspase activity is tightly regulated at multiple levels. Activation typically requires assembly of supramolecular signaling platforms, while endogenous inhibitor of apoptosis proteins (IAPs) modulate caspase stability and activity through ubiquitin-dependent degradation (Cabon et al., 2012; Dho et al., 2025). This multilayered regulation ensures that caspase activation occurs only under conditions of irreversible cellular damage or immune necessity, preventing accidental cell loss.

#### 5.2.3 Evolutive perspective

The caspase fold is evolutionarily ancient and widely distributed across all domains of life; however, bona fide apoptotic caspases, defined by strict aspartate specificity and adaptor-mediated activation, emerged exclusively within metazoans (Hengartner, 2000; Bibo-Verdugo and Salvesen, 2024). Phylogenomic analyses indicate that apoptotic caspases diversified early during animal evolution, accompanying the emergence of multicellularity and the need for regulated elimination of damaged or infected cells (Krasovec et al., 2023; Nadendla et al., 2025).

Recent evolutionary reconstructions suggest that caspase-2 is among the most ancestral bilaterian caspases, whereas caspases involved in death receptor signaling, such as caspase-8, show lineage-specific expansion and integration with immune pathways (Horkan et al., 2024; Prasanth Kumar et al., 2025; Zeng et al., 2024). Effector caspases within the caspase-3/6/7 subfamily display distinct biophysical and regulatory properties, reflecting progressive refinement of apoptotic execution mechanisms over more than 600 million years of evolution (Fan et al., 2005; Eskandari et al., 2022; Xu et al., 2024).

Collectively, these observations support the view that caspases evolved as a modular and adaptable proteolytic system, integrating apoptosis and inflammation to balance tissue homeostasis, immune defense, and host–pathogen interactions (Dho et al., 2025; Nadendla et al., 2025).

The dual involvement of caspases in apoptosis and inflammation places them at the core of host–pathogen interactions. Viruses, bacteria, fungi, protozoa, and helminths recurrently target caspase activation platforms, substrate specificity, or upstream adaptor complexes to either suppress apoptotic elimination or redirect cell death toward inflammatory outcomes that favor pathogen survival or dissemination. This selective pressure has contributed to the functional diversification, regulatory redundancy, and pathway integration of caspases observed across metazoans, setting the stage for the pathogen-specific strategies discussed in the following sections (Aubrey et al., 2018; Lamkanfi and Dixit, 2010; Rodríguez-González and Gutiérrez-Kobeh, 2023; Nadendla et al., 2025).

### 5.3 BCL-2: an ancient family of apoptotic proteins

Permeabilization of the mitochondrial outer membrane (MOMP) is the irreversible commitment step of intrinsic apoptosis and is controlled by the Bcl-2 family of proteins (Czabotar et al., 2014). Recent studies indicate that MOMP is a graded process, in which the threshold and timing depend on the balance between pro- and anti-apoptotic Bcl-2 members and the cellular stress context (Ichim et al., 2015). The family is named after BCL2, originally identified in B-cell leukemia/lymphoma (Tsujimoto et al., 1984). Bcl-2 proteins share one or more Bcl-2 homology (BH) domains (BH1–BH4) and act as either pro- or anti-apoptotic regulators, with cell fate determined by protein levels, binding interactions, localization, and post-translational modifications (Banjara et al., 2020).

Anti-apoptotic proteins such as Bcl-2, Bcl-xL, Mcl-1, and A1 preserve mitochondrial integrity by binding pro-apoptotic partners. In contrast, Bax and Bak oligomerize in the outer mitochondrial membrane to induce MOMP, while BH3-only proteins act as stress sensors that activate Bax/Bak or inhibit pro-survival Bcl-2 proteins. Structural studies published in 2025 show that BH3-mediated interactions promote conformational changes in Bax and Bak that allow progressive pore formation rather than fixed channels (Zhang et al., 2025; Jeng et al., 2018).

Evolutionary analyses indicate that the Bcl-2 family originated from a pro-apoptotic ancestor and diversified early during metazoan evolution (Popgeorgiev et al., 2020; Krasovec et al., 2023). Recent reconstructions suggest that pathogen-driven selection contributed to the expansion of antagonistic Bcl-2 paralogs, increasing the robustness of mitochondrial apoptosis against interference (Mustafa et al., 2024; Palmer et al., 2021). Despite divergence across taxa, the BH3 domain remains highly conserved, highlighting its central role in mitochondrial control of apoptosis.

Bid plays a linking role between extrinsic, granzyme-mediated, and intrinsic apoptotic pathways. After cleavage by caspase-8 or granzyme B, truncated Bid (tBid) translocates to mitochondria and promotes Bax/Bak activation. This function supports apoptosis when individual pathways are partially compromised, including during pathogen-mediated inhibition (Kaufmann et al., 2012; Jorgensen et al., 2017; Qi et al., 2021).

Across metazoans, the Bcl-2 repertoire varies. BH3-only proteins are absent in early-branching lineages, suggesting that they emerged later as refinements of intrinsic apoptosis. In *C. elegans*, apoptosis relies on a simplified system in which CED-9 regulates the APAF-1 homolog CED-4 independently of cytochrome c. In early chordates such as *Branchiostoma floridae*, expanded but non-redundant Bcl-2 repertoires indicate that antagonistic control of MOMP predates vertebrate genome duplications (Aouacheria et al., 2013; Jorgensen et al., 2017).

## 6 Apoptosis inhibition

In addition to Bcl-2 family–mediated regulation of mitochondrial integrity, apoptosis is tightly controlled by inhibitor of apoptosis proteins (IAPs), an evolutionarily conserved family of proteins present across eukaryotes. In mammals, eight IAPs (BIRC1–BIRC8), including NAIP, cIAP1, cIAP2, and XIAP, have been identified. All IAPs contain baculoviral IAP repeat (BIR) domains that mediate protein–protein interactions, while several also harbor a C-terminal RING domain conferring E3 ubiquitin ligase activity, enabling regulation of apoptotic and inflammatory signaling through ubiquitination (Fulda, 2018; Silke and Meier, 2013; Ye et al., 2025).

IAPs suppress apoptosis by binding to and ubiquitinating caspases or components of death receptor signaling complexes. cIAP1 and cIAP2 ubiquitinate RIPK1 downstream of TNF receptors, promoting NF-κB activation and preventing caspase-8–dependent apoptosis (Dumétier et al., 2020). Recent studies emphasize that this checkpoint is dynamically regulated by stress-activated pathways, particularly p38 MAPK, which fine-tunes TNF-driven survival versus apoptotic outcomes (Silke, 2011; Fernández-Lázaro et al., 2024; Desideri et al., 2025).

Apoptosis inhibition is further reinforced by pro-survival PI3K/Akt signaling. Akt preserves mitochondrial integrity and suppresses pro-apoptotic factors such as BAD and FoxO transcription factors, thereby limiting death-associated gene expression (Manning and Toker, 2017; Loureiro et al., 2023). Recent integrative analyses highlight extensive crosstalk between Akt, p38 MAPK, and IAPs, positioning these pathways as central coordinators of cell fate under inflammatory and genotoxic stress (Whitaker and Cook, 2021; Galluzzi et al., 2024).

Emerging evidence from 2025 further supports that intracellular pathogens and cancer cells converge on IAP-, Akt-, and p38-dependent signaling to suppress apoptosis and promote survival, underscoring apoptosis inhibition as an evolutionarily selected strategy shaped by chronic host–pathogen and tumor–host interactions (Ye et al., 2025; Szperlinski et al., 2025; Hu et al., 2025).

## 7 The other side of the coin: how pathogenic microorganisms modulate apoptosis to survive in host cells

Host–pathogen interactions are shaped by continuous coevolution, often described as Red Queen dynamics, in which both hosts and pathogens must constantly adapt to maintain fitness (Rabajante et al., 2015). Within this framework, apoptosis constitutes a central regulatory node, as its activation or suppression can decisively influence infection outcome.

Cells of the innate immune system, particularly phagocytes, rely on multiple regulated cell death programs as antimicrobial defenses. In response, pathogens have evolved mechanisms to interfere with these pathways at several levels, including death receptor signaling, DISC assembly, mitochondrial permeabilization, and transcriptional regulation of survival genes. Viruses provide clear examples of this coevolutionary conflict, frequently inhibiting apoptosis during early replication and inducing it at later stages to promote viral release and immune evasion (Kvansakul and Hinds, 2013).

Apoptosis also contributes to host defense by limiting pathogen replication and enabling the immunologically silent clearance of infected cells through efferocytosis. Recognition of apoptotic “eat-me” signals, such as phosphatidylserine, promotes removal of dying cells while minimizing tissue damage and excessive inflammation (Behar and Briken, 2019; Naderer and Fulcher, 2018). However, several pathogens exploit this process by modulating apoptotic signaling or altering ubiquitin-dependent regulatory checkpoints, thereby balancing cell survival and death in favor of persistence.

### 7.1 Viral modulation of apoptosis: evolutionary strategies for intracellular survival

Viruses are obligate intracellular pathogens that depend entirely on host cell machinery for replication. As a countermeasure, host cells deploy apoptosis as an early antiviral defense to eliminate infected cells and limit viral spread (Aubrey et al., 2018). This antagonistic interaction has driven a long-standing host–virus arms race, in which viruses have evolved convergent strategies to modulate apoptotic signaling in a temporally controlled manner. A common viral strategy is to suppress apoptosis during early infection to preserve host cell viability and enable genome replication, followed by apoptosis induction at later stages to facilitate viral egress and dissemination. Molecular mimicry is central to this process, as many viruses encode functional analogs of host apoptotic regulators. For example, *Ectromelia virus*, *Poxviridae* (mousepox) expresses soluble TNF receptor decoys (CrmB–E) that neutralize TNF ligands and block extrinsic apoptotic signaling, while several herpesviruses, including Epstein–Barr virus (Human gammaherpesvirus 4) and Murine gammaherpesvirus 68, as well as poxviruses such as Vaccinia virus, encode viral FLIP (vFLIP) proteins that inhibit caspase-8 activation at the death-inducing signaling complex (DISC) (Aubrey et al., 2018; Yu and Damania, 2024; Hernaez and Alcamí, 2024).

Beyond receptor-proximal events, viruses converge on conserved apoptotic nodes downstream of death receptor signaling. Hepatitis B virus (Hepatitis B virus, family Hepadnaviridae), human immunodeficiency virus type 1 (Human immunodeficiency virus 1, Retroviridae), and multiple herpesviruses, including Human cytomegalovirus (Human betaherpesvirus 5), interfere with Fas- or TRAIL-mediated apoptosis. In parallel, poxviruses such as Vaccinia virus and Ectromelia virus produce serpins (e.g., CrmA) that inhibit caspases involved in both apoptosis and inflammation. Comparative analyses across these viral species reveal that apoptotic inhibition is frequently coordinated with modulation of additional cell death pathways, including necroptosis and autophagy, highlighting integrated viral control of host cell fate (Suehiro et al., 2023; Vlachava et al., 2023; Nourazarian et al., 2025).

Another frequent target is p53, a central transcriptional regulator of apoptosis. Multiple viruses, including Human papillomavirus (high-risk types such as HPV-16), Epstein–Barr virus, and Human cytomegalovirus, destabilize or repress p53 activity through viral proteins or viral microRNAs, thereby preventing transcription of pro-apoptotic genes such as PUMA, NOXA, FAS, and APAF1 (Wang et al., 2023). Recent comparative studies indicate that p53 suppression often cooperates with mitochondrial checkpoint regulation across diverse viral species, reinforcing inhibition of intrinsic apoptosis during productive infection (Bakhanashvili, 2024; Dong et al., 2024).

Mitochondrial apoptosis represents a remarkably conserved viral target. Numerous viruses encode Bcl-2 homologues or Bcl-2-like proteins that inhibit mitochondrial outer membrane permeabilization by sequestering BH3-only proteins or Bax/Bak. Classic examples include adenoviral E1B-19K from Human adenovirus C, BHRF1 from Epstein–Barr virus, and M11 from Murine gammaherpesvirus 68, the latter also suppressing autophagy through Beclin-1 binding (Cuconati and White, 2002; Ramanathan et al., 2020).

Recent studies on SARS-CoV-2 illustrate how contemporary viruses exploit these conserved mechanisms. Severe acute respiratory syndrome coronavirus 2 (SARS-CoV-2) induces apoptosis through its ORF3a protein by activating caspase-8 and promoting Bid cleavage, thereby engaging the extrinsic apoptotic pathway and mitochondrial amplification. Experimental evidence from human epithelial and lung-derived cell models demonstrates that ORF3a-mediated apoptosis during SARS-CoV-2 infection is associated with mitochondrial depolarization and ion channel dysregulation, reinforcing the role of mitochondrial checkpoints in coronavirus-induced cell death (Qudus et al., 2025). Comparative analyses with severe acute respiratory syndrome coronavirus (SARS-CoV) further suggest involvement of p38 kinase and p53-dependent pathways, although virus-specific differences remain to be fully defined (Minakshi and Padhan, 2014; Mittal et al., 2024).

**FIGURE 3:**
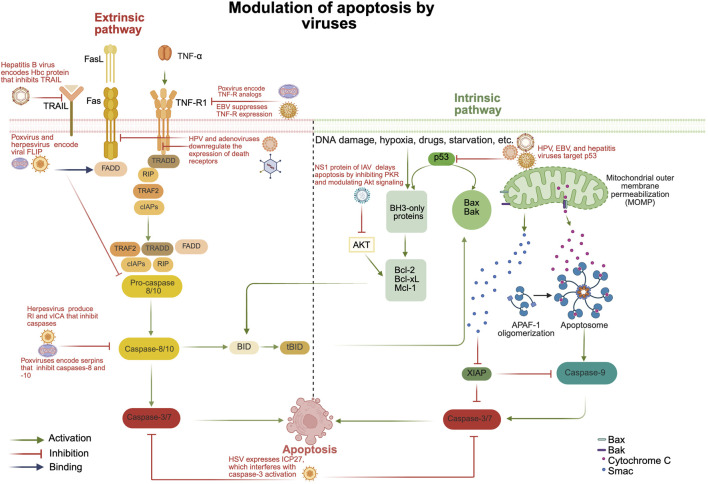
Viral modulation of extrinsic and intrinsic apoptotic pathways. This schematic illustrates how viruses interfere with host cell apoptosis at multiple regulatory checkpoints. In the extrinsic pathway, viral proteins inhibit death receptor signaling by targeting Fas, TNF-R1, FADD, and initiator caspases, thereby preventing caspase-8/10 activation and downstream executioner caspases. In the intrinsic pathway, several viruses modulate p53 activity, alter Bcl-2 family dynamics, and interfere with mitochondrial outer membrane permeabilization (MOMP), limiting cytochrome c release and apoptosome formation. Additional viral strategies include activating survival signaling via AKT, inhibiting apoptosome function, and directly blocking caspase activity. Together, these mechanisms allow viruses to fine-tune host cell fate to favor viral replication and persistence.

Collectively, viral targeting of caspase-8, Bid-mediated mitochondrial amplification, and the Bax/Bak checkpoint across unrelated viral families, including Poxviridae, Herpesviridae, Retroviridae, Hepadnaviridae, and Coronaviridae, highlights the deep evolutionary conservation of apoptotic control points. (Figure 3).

### 7.2 Bacterial control of host apoptosis: indirect and inflammatory strategies

During bacterial infection, host cells activate multiple forms of regulated cell death (RCD), including apoptosis, to restrict pathogen replication and dissemination. In response, bacteria have evolved strategies to either induce or suppress host cell death, depending on which outcome best supports their survival and transmission (Szperlinski et al., 2025; He et al., 2025).

Extracellular bacterial pathogens often promote inflammatory or lytic forms of cell death to facilitate escape and dissemination. Species such as *Serratia marcescens*, *Staphylococcus aureus*, and *Streptococcus pneumoniae* induce necroptosis in epithelial and endothelial cells through pore-forming toxins, including hemolysins and leukocidins, allowing bacterial release while avoiding sequestration into apoptotic bodies and subsequent efferocytic clearance (González-Juarbe et al., 2015; Kitur et al., 2016; Mei et al., 2024).

In contrast, many intracellular or facultative intracellular bacteria suppress apoptosis to preserve a viable intracellular niche. One indirect but widespread mechanism involves the release of outer membrane vesicles (OMVs), which deliver bacterial components that modulate host immune signaling. OMVs derived from *Neisseria gonorrhoeae*, *Escherichia coli*, and *Pseudomonas aeruginosa* activate the NLRP3 inflammasome, leading to caspase-1 activation, gasdermin D cleavage, and pyroptosis (Lamkanfi and Dixit, 2010; He and Amer, 2014). Recent work demonstrates that OMVs can simultaneously trigger pyroptosis in immune cells while suppressing mitochondrial apoptosis in epithelial cells, generating spatially compartmentalized cell death responses that favor bacterial persistence and inflammation-driven dissemination (Sabatke et al., 2025).

Bacterial infections frequently converge on mitochondrial control of apoptosis. Toxins and metabolic stressors disrupt mitochondrial homeostasis, downregulate anti-apoptotic Bcl-2 family members such as Mcl-1, and promote Bak-mediated mitochondrial outer membrane permeabilization (MOMP) and caspase-dependent apoptosis. Mitochondrial fragmentation, impaired mitophagy, and redox imbalance have emerged as key intermediates linking bacterial virulence factors to apoptotic commitment (Maurice and Sadikot, 2023; Harishankar and Viswanathan, 2023). Particularly, several bacterial virulence factors directly target mitochondria and conduct to apoptosis. The VacA toxin of *Helicobacter pylori* induces apoptosis in gastric epithelial cells via mitochondrial disruption (Chiozzi et al., 2009). *Vibrio cholerae* cytolysin (VCC), a β-barrel pore-forming toxin, and its pore-deficient mutants translocate to mitochondria and induce membrane damage (Kaur et al., 2022). Similarly, the *V. cholerae* porin OmpU promotes programmed cell death through mitochondrial dysfunction (Gupta et al., 2015). In *N. gonorrhoeae*, the porin PorB is delivered to host mitochondria via outer membrane vesicles (OMVs), leading to loss of mitochondrial membrane potential, cytochrome c release, caspase activation, and time-dependent apoptosis (Deo et al., 2018). Enteropathogenic and enterohemorrhagic *E. coli* (EPEC and EHEC) utilize a type III secretion system (T3SS) to inject effectors that modulate host cell death pathways. While bacterial surface components can activate extrinsic apoptosis (Abul-Milh et al., 2001), effectors such as EspF and Map predominantly trigger intrinsic pathways. EspF induces mitochondrial dysfunction and promotes degradation of anti-apoptotic proteins, whereas Map impairs mitochondrial function (Kenny and Jepson, 2000; Ma et al., 2006; Nougayrède and Donnenberg, 2004).

Host metabolic state further modulates apoptotic sensitivity during bacterial infection. In murine models of *E. coli* lung infection, obesity-associated endoplasmic reticulum stress enhances TNF-α/TNFR1-mediated apoptosis, exacerbating tissue injury and impairing bacterial clearance (Wang et al., 2020). Recent studies extend these findings by demonstrating that metabolic inflammation amplifies death receptor–mediated apoptosis during Gram-negative infections (Song et al., 2019; Wang et al., 2020).

**FIGURE 4:**
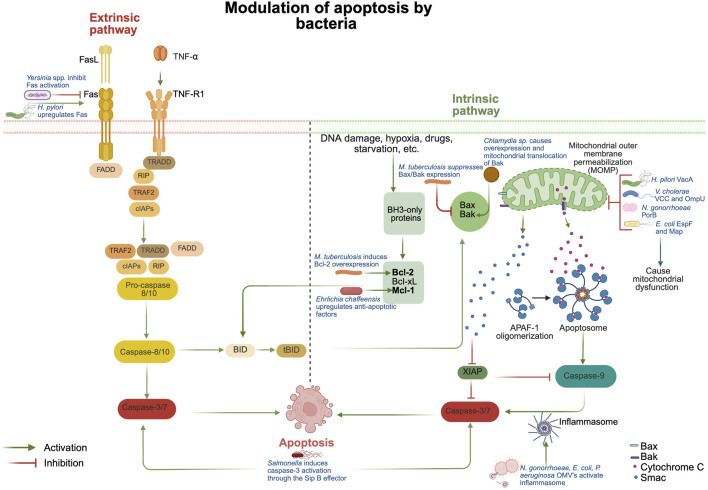
Modulation of host apoptosis by bacterial pathogens. Bacterial pathogens dynamically regulate host apoptotic pathways to optimize survival, replication, and dissemination. As illustrated, bacteria can either induce or inhibit apoptosis depending on their lifestyle and stage of infection. Extracellular pathogens commonly activate death receptor signaling via Fas and TNF receptors, leading to caspase-8 activation and downstream engagement of executioner caspases. In contrast, many intracellular bacteria suppress mitochondrial apoptosis by modulating the balance between pro- and anti-apoptotic Bcl-2 family proteins, thereby preserving host cell viability and maintaining a permissive intracellular niche. Multiple pathogens converge on mitochondrial control by regulating Bax and Bak activation, mitochondrial outer membrane permeabilization (MOMP), cytochrome c release, and apoptosome assembly. In addition, certain bacterial virulence factors directly impair mitochondrial function, ultimately promoting apoptosis. Others indirectly influence host cell fate through inflammatory signaling and inflammasome activation, generating spatially compartmentalized cell death responses that support bacterial persistence and inflammation-driven dissemination. Collectively, these mechanisms position apoptosis as a central regulatory hub targeted by bacterial virulence strategies in a highly context- and stage-dependent manner.

A paradigmatic example of bacterial modulation of apoptosis is provided by *Chlamydia* species, particularly *Chlamydia trachomatis*, which exert stage-dependent control over host cell death. While early studies reported caspase-independent apoptosis associated with Bax translocation (Sixt, 2021), later work demonstrated that *C. trachomatis* actively suppresses mitochondrial apoptosis during early replication by inhibiting cytochrome c release and Bax activation (Kontchou et al., 2022). Recent single-cell and temporal analyses confirm that apoptotic and lytic pathways are selectively reactivated during late stages of infection, coinciding with bacterial egress (Yang et al., 2024) (Figure 4).

### 7.3 Modulation of host cell apoptosis by pathogenic fungi

Pathogenic fungi actively modulate apoptosis in host cells to promote survival, tissue invasion, and immune evasion. During infections with *Candida albicans*, *Cryptococcus neoformans*, and *Aspergillus fumigatus*, host cell apoptosis is often delayed or suppressed, particularly in macrophages, epithelial cells, and endothelial cells, allowing fungal persistence during the early stages of infection (Ibata-Ombetta et al., 2003; Shlezinger et al., 2017; Zhang et al., 2021; Williams et al., 2021).

Several fungal pathogens interfere with intrinsic apoptotic signaling in host cells, including modulation of mitochondrial apoptotic checkpoints and downstream caspase activation. In macrophages infected with *C. albicans*, altered regulation of mitochondrial apoptosis and reduced activation of executioner caspases correlate with enhanced fungal survival, indicating direct interference with host intrinsic death pathways (Ibata-Ombetta et al., 2003; Wellington et al., 2014). Similarly, *C. neoformans* modulates host mitochondrial function and oxidative stress responses to restrain apoptosis and favor intracellular persistence (Coelho et al., 2015; Zhang et al., 2021).

Stress-activated signaling pathways are central to this process. Fungal components activate host MAPK and PI3K/Akt signaling pathways, which promote pro-survival and inflammatory responses while restraining apoptotic execution. In host cells exposed to *A. fumigatus*, sustained activation of p38 MAPK is associated with enhanced stress tolerance and delayed apoptotic responses (Shlezinger et al., 2011; Shlezinger et al., 2017), while PI3K/Akt signaling contributes to mitochondrial stabilization and suppression of pro-apoptotic factors during fungal infection (Williams et al., 2021).

Overall, modulation of host cell apoptosis by pathogenic fungi represents a conserved strategy that enhances fungal fitness under immune pressure. By targeting mitochondrial checkpoints and stress-response pathways, fungi delay host cell death while maintaining inflammatory environments that support persistence and dissemination, consistent with broader patterns of host–pathogen coevolution (Williams et al., 2021; Kaczanowski, 2016).

### 7.4 Parasitic interactions and the evolutionary plasticity of apoptotic pathways

Host–parasite interactions reflect a long-standing coevolutionary arms race in which apoptosis emerged as a host defense mechanism to eliminate infected cells, while parasites evolved strategies to suppress, redirect, or exploit regulated cell death to enhance survival and transmission (Kaczanowski, 2016). Because core apoptotic pathways predate complex multicellularity, many parasites target deeply conserved mitochondrial and caspase-associated nodes, particularly mitochondrial integrity, Bcl-2 family balance, and caspase activation.

#### 7.4.1 Stage-specific modulation of host apoptosis by protozoan parasites

Protozoan parasites such as *Leishmania*, *Trypanosoma*, and *Plasmodium* exhibit highly stage-specific control of host apoptosis. Rather than uniformly inducing or inhibiting cell death, these organisms dynamically modulate apoptotic pathways in response to developmental requirements. *Leishmania major*, for example, expresses the metalloprotease GP63, which cleaves host caspase-3 and associated signaling proteins, thereby suppressing macrophage apoptosis and promoting intracellular persistence (Samanta et al., 2025). *Plasmodium falciparum* suppresses mitochondrial apoptosis during early hepatocyte infection but later activates apoptotic pathways to facilitate merozoite release and dissemination (van de Sand et al., 2005). Similarly, trypanosomatids interfere with Bcl-2-regulated mitochondrial checkpoints, limiting cytochrome *c* release and attenuating intrinsic apoptosis (Román-Carraro et al., 2022). Particularly, infection of macrophages with *L. major* or *L. donovani* promastigotes induces the overexpression of the anti-apoptotic proteins Bcl-xL, Bcl-2, and Bfl-1 (Donovan et al., 2009; Sarkar et al., 2013).

**FIGURE 5:**
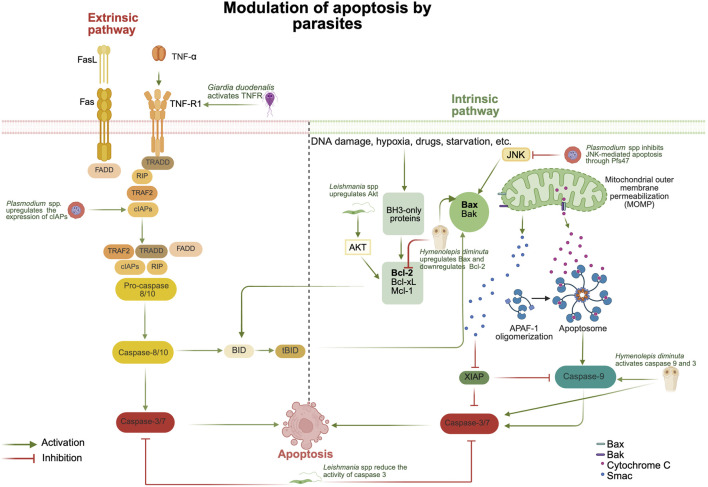
Parasite-mediated regulation of extrinsic and intrinsic apoptotic pathways during host–pathogen interactions. The extrinsic apoptotic pathway is initiated by the engagement of death receptors, including Fas and TNFR, leading to the recruitment of adaptor proteins such as FADD and TRADD, activation of RIP and TRAF2, and subsequent processing of initiator caspases-8 and -10. Activated caspase-8 directly triggers executioner caspases-3 and -7 and connects to the intrinsic pathway through cleavage of BID into tBID. The intrinsic pathway is activated by intracellular stress signals, including DNA damage, hypoxia, nutrient deprivation, and inflammatory cues, which promote the activation of BH3-only proteins and the balance between pro-apoptotic (Bax/Bak) and anti-apoptotic (Bcl-2, Bcl-xL, Mcl-1) Bcl-2 family members. Bax/Bak-mediated mitochondrial outer membrane permeabilization (MOMP) enables cytochrome c release, apoptosome assembly, and caspase-9 activation, culminating in executioner caspase activation and apoptosis. Crosstalk between pathways is tightly regulated by survival signaling cascades such as PI3K/Akt and stress-activated kinases (e.g., JNK), as well as by inhibitor of apoptosis proteins (IAPs), including XIAP. During infection, diverse pathogens—including bacteria, fungi, protozoa, and helminths—target multiple nodes within these pathways to either promote or restrain host cell death, thereby shaping immune responses, tissue damage, repair, and pathogen persistence.

A recurring feature of protozoan infection is temporal control of apoptosis. During early stages, parasites upregulate host anti-apoptotic proteins such as Bcl-2, Bcl-xL, and Mcl-1, while activating survival pathways including NF-κB, PI3K/Akt, and MAPK. At later stages, apoptosis may be actively induced to promote parasite egress, tissue dissemination, or immune evasion (Coria-Paredes et al., 2025). This regulation is also markedly species-dependent within the same genus. In macrophages infected *in vitro*, *Leishmania amazonensis*, but not *L. guyanensis*, induces a classical apoptotic program characterized by phosphatidylserine exposure, nucleosomal DNA fragmentation, and activation of caspases-3, -8, and -9 as early as 4 h post-infection, supporting the idea that apoptosis can be triggered at specific early windows to favor chronic infection and parasite spreading through uptake of apoptotic infected cells by neighboring macrophages. These findings reinforce that protozoan control of host cell death is not only stage-specific but also species-specific and may determine whether apoptosis functions as a survival strategy, a dissemination mechanism, or a pathogenicity-associated trait (DaMata et al., 2015) (Figure 5).

#### 7.4.2 Apoptosis-like cell death pathways in unicellular parasites

Several unicellular parasites display apoptosis-like cell death programs characterized by mitochondrial depolarization, DNA fragmentation, and caspase-like protease activity, despite lacking canonical metazoan caspases (Böttger and Alexandrova, 2007; Duszenko et al., 2006). These pathways appear to regulate parasite population density, stress adaptation, and life-cycle transitions, supporting the view that regulated cell death represents a deeply conserved eukaryotic trait that predates metazoan apoptosis and was later elaborated in multicellular organisms (Ameisen, 2002; Lockshin and Zakeri, 2004). Additionally, apoptotic cells and apoptosis-derived molecules are well recognized for their role in the establishment and progression of several parasitic infections. This mechanism was first demonstrated in 2001, when amastigote forms of *L. amazonensis* were shown to expose phosphatidylserine (PS) on the outer leaflet of their plasma membrane. This exposure was found to be critical for the infection of host cells by amastigotes and for the maintenance of the resulting experimental leishmanial disease (Wanderley et al., 2006).

#### 7.4.3 Helminth-mediated suppression of apoptosis and systemic immunomodulation

In contrast to protozoa, helminths primarily modulate apoptosis at the tissue and systemic level rather than inducing direct cytolysis. Helminths such as *Schistosoma mansoni*, *Heligmosomoides polygyrus*, and *Brugia malayi* secrete extracellular vesicles, microRNAs, and protease inhibitors that enhance host expression of anti-apoptotic proteins and suppress caspase activation, thereby promoting immune tolerance, tissue repair, and chronic infection (Maizels et al., 2018; Drurey and Maizels, 2021).

Recent studies demonstrate that helminth-derived extracellular vesicles (EVs) from *S. mansoni* and *H. polygyrus* are internalized by macrophages, dendritic cells, and intestinal epithelial cells, where they upregulate anti-apoptotic proteins such as Bcl-2, Bcl-xL, and survivin while downregulating caspase-3 and caspase-9 activation (Eichenberger et al., 2018). These effects preserve host cell viability and contribute to long-term parasite persistence.

Beyond extracellular vesicle–mediated effects, helminths can modulate death-receptor signaling in a cell- and context-dependent manner rather than simply inducing or suppressing apoptosis. During *Schistosoma japonicum* infection, egg-derived antigens engage death-receptor–associated pathways in the liver, particularly in hepatic stellate cells, where DR5/TRAIL-related signaling intersects with fibrogenic remodeling during chronic disease (Wang et al., 2014; Yu et al., 2024). In parallel, murine models show that hepatocytes undergo cellular stress accompanied by increased apoptotic susceptibility, indicating that apoptosis regulation during infection varies across hepatic cell populations and stages of infection (Yu et al., 2024).

Similarly, *Fasciola hepatica* releases excretory–secretory molecules and extracellular vesicles that primarily reprogram hepatic immune responses instead of directly triggering host cell death. These parasite-derived factors promote regulatory and anti-inflammatory states in liver-resident immune cells, thereby shaping a tissue environment that supports parasite persistence (Murphy et al., 2020; Bąska et al., 2024).

Helminth-mediated modulation of apoptosis is tightly linked to their ability to skew host immunity toward type 2 and regulatory responses. Infections with *H. polygyrus* and *Nippostrongylus brasiliensis* promote IL-4/IL-13–driven alternative macrophage activation, which is associated with enhanced cellular survival programs and increased resistance to pro-apoptotic signaling, contributing to tissue protection during chronic infection (Gause et al., 2013; Maizels and Gause, 2023). This close coupling of immune polarization and controlled regulation of cell death represents a hallmark of helminth–host coadaptation.

Importantly, helminth-driven regulation of apoptosis extends beyond immune cells to structural tissues. In intestinal and pulmonary infection models, helminths such as *Trichuris muris* and *N. brasiliensis* enhance epithelial cell survival and wound-healing programs by limiting excessive apoptosis while promoting epithelial turnover, thereby maintaining barrier integrity despite persistent infection (Cliffe et al., 2007; Rolot and Dewals, 2018).

From an evolutionary perspective, this modulation of apoptosis reflects a strategy optimized for long-term persistence rather than rapid dissemination. By restraining excessive host cell death while promoting tissue repair and immune tolerance, helminths establish stable host–parasite equilibria that can persist for years or decades. This strategy contrasts with the acute cytolytic or inflammatory cell death programs frequently favored by viruses and bacteria, underscoring apoptosis as a flexible, context-dependent target shaped by distinct evolutionary pressures (Maizels and McSorley, 2016).

### 7.5 Pathogen-mediated modulation of the p38 MAPK pathway

The p38 MAPK pathway plays a central role in host responses to infection by integrating stress, inflammatory, and apoptotic signals. Because of this position, p38 is frequently modulated by pathogens to influence host cell fate. Rather than simply inducing or blocking apoptosis, pathogen-mediated regulation of p38 adjusts the balance between immune activation, cell survival, and cell death in ways that favor infection while limiting excessive tissue damage (Cuadrado and Nebreda, 2010; Arthur and Ley, 2013; Gräb and Rybniker, 2019).

Several bacterial pathogens exploit p38 signaling during infection. *Salmonella enterica* activates p38 MAPK to promote inflammatory gene expression while delaying apoptosis of infected epithelial cells. In macrophages infected with *Mycobacterium tuberculosis*, sustained p38 activation supports cytokine production and restricts apoptotic pathways, contributing to intracellular survival and granuloma formation. *Listeria monocytogenes* similarly engages p38 to induce inflammatory responses and stress-associated apoptosis, emphasizing its role as a regulator of host cell viability during bacterial infection (Tang et al., 1998; Cuadrado and Nebreda, 2010; Arthur and Ley, 2013).

Viral pathogens also target p38 MAPK to optimize replication. Influenza A virus requires p38 activation for efficient replication and induces inflammatory and apoptotic responses in epithelial cells. In contrast, herpesviruses such as Epstein–Barr virus and cytomegalovirus activate p38 while interfering with downstream apoptotic execution, allowing infected cells to remain viable during viral replication. This uncoupling of stress signaling from cell death illustrates a common viral strategy (Nencioni et al., 2009; Börgeling et al., 2014; Meineke et al., 2019).

Protozoan parasites further highlight the flexibility of p38 modulation. In *L. major–*infected macrophages, p38 activation contributes to cytokine production while apoptotic responses are limited by parasite-derived mechanisms. Also, *Leishmania mexicana* amastigotes and promastigotes downregulate p-38 as a mechanism to inhibit dendritic cells apoptosis (Rodríguez-González et al., 2016; Vázquez-López et al., 2015). During *Trypanosoma cruzi* infection, p38 is activated by oxidative and inflammatory stress and influences immune signaling and apoptosis in macrophages and cardiomyocytes, shaping chronic infection and tissue pathology rather than rapid cell elimination (Mukherjee et al., 2004; Soares-Silva et al., 2016; Solano-Gálvez et al., 2021).

Recent integrative studies suggest that p38 MAPK functions as a convergence hub for pathogen-induced metabolic, oxidative, and inflammatory stress, coordinating not only apoptotic decisions but also broader programs of regulated cell death and immune adaptation. In this context, p38 signaling operates as a dynamic rheostat rather than a binary switch, enabling pathogens to fine-tune host cell fate in a manner that promotes persistence while avoiding premature immune clearance (Gräb and Rybniker, 2019; Shabarnia et al., 2023).

Together, these examples indicate that p38 MAPK is a conserved signaling node repeatedly targeted by pathogens to control host responses. By modulating p38 activity, pathogens influence whether infected cells survive, undergo apoptosis, or sustain inflammatory signaling, supporting the view that p38-dependent apoptotic regulation is embedded within broader infection-driven stress and immune networks shaped by host–pathogen coevolution.

### 7.6 Pathogen pressure and the evolution of core apoptotic regulators

Pathogen pressure has been a significant force shaping the evolution and regulatory refinement of core apoptotic components, particularly the Bcl-2 family, Bid, and Apaf-1. Rather than evolving solely for development and tissue homeostasis, apoptotic pathways were repeatedly remodeled by infection, resulting in increased redundancy, pathway integration, and robustness.

A first line of evidence comes from the expansion and functional diversification of the Bcl-2 family, which reflects sustained selection imposed by pathogens that recurrently target mitochondrial outer membrane permeabilization (MOMP). Viral Bcl-2 homologs encoded by Epstein–Barr virus, Kaposi’s sarcoma–associated herpesvirus, and poxviruses, together with parasitic suppression of mitochondrial apoptosis by *Leishmania* and *Trypanosoma* species, favored the retention of antagonistic paralogs and enhanced regulatory control over mitochondrial apoptosis (Cuconati and White, 2002; Kaczanowski, 2016). Phylogenomic analyses further show that lineage-specific expansions of Bcl-2 family members correlate with pathogen diversity and infection burden across metazoans, supporting pathogen-driven selection on mitochondrial regulation of apoptosis (Suraweera et al., 2024; Horkan et al., 2024). Together, these findings support a model in which mitochondrial apoptosis has been repeatedly targeted during infection, driving the retention of functionally antagonistic Bcl-2 paralogs and promoting a regulatory architecture that balances robustness with plasticity in the face of pathogen interference (Cuconati and White, 2002; Galluzzi et al., 2012; Horkan et al., 2024).

A second example is the emergence and conservation of Bid as an atypical BH3-only protein with a Bcl-2-like fold. Bid integrates death receptor signaling, cytotoxic lymphocyte activity, and mitochondrial apoptosis, allowing host cells to bypass pathogen-mediated blockade of individual pathways (Aubrey et al., 2018; Strasser and Vaux, 2020). Recent infection models demonstrate that Bid-dependent mitochondrial amplification restores apoptotic competence when upstream signaling is inhibited, reinforcing its role as an evolutionary integrator of apoptotic pathways (Pasparakis and Vandenabeele, 2015).

Finally, pathogen pressure has also shaped Apaf-1 and apoptosome-dependent caspase activation. Numerous viruses and intracellular bacteria inhibit initiator caspases or upstream adaptors, promoting diversification of regulatory checkpoints within the intrinsic pathway (Best, 2008; Friedrich et al., 2017). Comparative structural analyses reveal adaptive variation in Apaf-1 domains targeted by viral inhibitors, consistent with ongoing host–pathogen arms races at the level of apoptosome assembly. These observations indicate that the apoptosome represents an additional evolutionary hotspot repeatedly sculpted by pathogen interference, further reinforcing the view that core apoptotic regulators have not evolved in isolation but under continuous infectious pressure (Yuan and Akey, 2013; Mustafa et al., 2024).

Pathogenic fungi provide additional support for this model. Although fungi lack canonical Bcl-2 family proteins and caspases, pathogens such as *C. albicans*, *C. neoformans*, and *A. fumigatus* efficiently modulate host apoptosis by targeting conserved mitochondrial and stress-responsive checkpoints. By stabilizing mitochondrial integrity and activating host survival pathways, including p38 MAPK and PI3K/Akt signaling, fungi delay host cell death and promote intracellular survival, reinforcing mitochondrial apoptosis as a central and conserved target of pathogen pressure (Shlezinger et al., 2017; Friedrich et al., 2017; Tummers and Green, 2022).

Taken together, evidence from viruses, bacteria, protozoa, and fungi converges on a unifying evolutionary principle: mitochondrial apoptosis and its regulatory nodes constitute a restricted set of conserved vulnerabilities that have been repeatedly exploited by pathogens and, in turn, refined by host selection (Kulkarni et al., 2019; Daskalov, 2025).

## 8 Concluding remarks: apoptosis as an evolutionary battleground

Apoptosis emerges as a fundamental biological process whose evolution cannot be understood without considering the persistent pressure imposed by pathogens. Across viruses, bacteria, parasites, and fungi, diverse pathogens converge on a limited set of conserved apoptotic nodes, particularly MOMP, Bcl-2 family regulation, caspase activation, and integrative modules such as Bid and Apaf-1. The recurrent targeting of these checkpoints underscores their ancient origin, functional centrality, and evolutionary robustness (Green and Llambi, 2015; Pasparakis and Vandenabeele, 2015; Suraweera et al., 2020).

At the same time, differences in apoptotic modulation strategies reflect distinct pathogen life-history constraints. Viruses often rely on direct molecular mimicry and inhibition of apoptotic regulators, bacteria predominantly modulate apoptosis indirectly through metabolic stress and inflammatory signaling, while parasites and fungi display greater regulatory plasticity, dynamically suppressing or delaying host cell death to support persistence and dissemination (Cuconati and White, 2002; Raju et al., 2012).

Significantly, pathogen-driven selection has not only shaped how apoptosis is deployed during infection but has also contributed to the expansion, redundancy, and regulatory complexity of apoptotic machinery itself. The diversification of Bcl-2 family members, the emergence of pathway-bridging proteins such as Bid, and the refinement of apoptosome-dependent caspase activation illustrate how apoptotic networks evolved increased modularity and resilience to counteract microbial subversion (Youle and Strasser, 2008; Kale et al., 2018; Tummers and Green, 2022).

Recent evolutionary syntheses further support the view that regulated cell death pathways, including apoptosis, have coevolved with microbial pathogens as integral components of innate immunity, positioning apoptotic signaling networks as both defensive barriers and recurrent targets of pathogen adaptation (Jorgensen et al., 2017; Tummers and Green, 2022; Fernández-Lázaro et al., 2024).

In line with this framework, contemporary consensus now recognizes apoptosis as part of a broader, integrated network of regulated cell death programs that operate as core components of host defense and immune surveillance (Galluzzi et al., 2024). In this context, apoptosis does not function as an isolated pathway, but as a central module within an evolutionarily conserved system of antimicrobial protection that has been repeatedly reshaped by pathogen-driven selective pressure (Jorgensen et al., 2017; Galluzzi et al., 2024; Mustafa et al., 2024; Tummers and Green, 2022).

**FIGURE 6:**
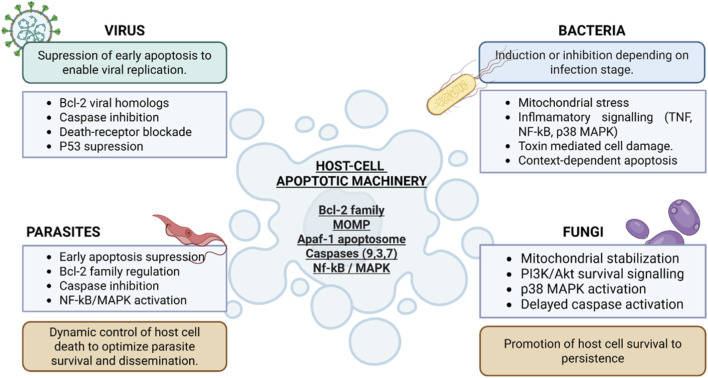
Pathogen-driven modulation of host apoptosis during infection. Apoptosis is a central cell-intrinsic defense mechanism that limits pathogen replication by eliminating infected cells. However, viruses, bacteria, fungi, and parasites have evolved convergent strategies to manipulate host apoptotic machinery to promote their own survival and dissemination. As illustrated, diverse pathogens target a restricted set of conserved regulatory nodes, including the Bcl-2 family, mitochondrial outer membrane permeabilization (MOMP), Apaf-1–dependent apoptosome assembly, caspase activation, and stress-activated signaling pathways such as NF-κB and MAPKs. Viruses commonly suppress early apoptosis through molecular mimicry and caspase inhibition to preserve host cell viability during replication. Bacterial pathogens modulate apoptosis in a context-dependent manner through mitochondrial stress, toxin-mediated damage, and inflammatory signaling. Pathogenic fungi delay host cell death by stabilizing mitochondrial integrity and activating pro-survival pathways, thereby favoring intracellular persistence. In contrast, protozoan parasites such as *Trypanosoma cruzi* dynamically regulate host apoptosis in a stage-dependent manner to optimize long-term survival and dissemination. Together, these interactions highlight apoptosis as an evolutionary battleground shaped by continuous host–pathogen coevolution.

Together, the evidence synthesized in this review supports a framework in which apoptosis represents an evolutionary battleground shaped by continuous host–pathogen coevolution, while also providing a rich source of conserved regulatory nodes with clear translational potential (Figure 6).
